# Healthcare providers’ knowledge, attitude, and practice on quality of nutrition care in hospitals from a developing country: a multicenter experience

**DOI:** 10.1186/s41043-023-00355-9

**Published:** 2023-03-07

**Authors:** Muna Shakhshir, Abdulsalam Alkaiyat

**Affiliations:** 1 Department of Nutrition, An-Najah National University Hospital, Nablus, 44839 Palestine; 2 Health Division, College of Medicine and Health Sciences, An-Najah National University, Nablus, 44839 Palestine

**Keywords:** Healthcare providers, Knowledge, Attitude, And practice, Nutrition care, Palestine

## Abstract

**Background:**

Despite the fact that malnutrition can affect both recovery and outcome in acute care patients, little is known about malnutrition in Palestine, and even less is known about the assessment of malnutrition knowledge, attitudes, and practices (M-KAP) toward healthcare providers and nutrition care quality measures in hospitalized patients**.** Therefore, this study aimed to evaluate the M-KAP of physicians and nurses in routine clinical care and determine the influencing factors.

**Methods:**

From April 1 to June 31, 2019, cross-sectional research was performed at governmental (*n* = 5) and non-governmental (*n* = 4) hospitals in the North West Bank of Palestine. Data were collected using a structured self-administered questionnaire from physicians and nurses to collect information on knowledge, attitude, and practices related to malnutrition and nutrition care, alongside sociodemographic characteristics.

**Results:**

A total of 405 physicians and nurses were participated in the study. Only 56% of participants strongly agreed that nutrition was important, only 27% strongly agreed that there should be nutrition screening, only 25% felt food helped with recovery, and around 12% felt nutrition as part of their job. Approximately 70% of participants said they should refer to a dietitian, but only 23% knew how and only 13% knew when. The median knowledge/attitude score was 71, with an IQR ranging from 65.00 to 75.00, and the median practice score was 15.00 with an IQR of 13.00–18.00. The mean knowledge attitude practice score was 85.62 out of 128 with SD (9.50). Respondents who worked in non-governmental hospitals showed higher practice scores (*p* < 0.05), while staff nurses and ICU workers showed the highest practice score (*p* < 0.001). Respondents with younger age categories, working in non-governmental hospitals in the ICU as practical and staff nurses, showed the highest KAP score (*p* < 0.05). Significance positive correlations were found between respondents’ knowledge/attitude and practice scores regarding the quality of nutrition care in hospitals (*r* = 0.384, *p* value < 0.05). In addition, the result also revealed that almost half of respondents believed that the most important barriers to inadequate intake of food at the bedside are related to food appearance, taste, and aroma of meals served (58.0%).

**Conclusions:**

The research revealed that inadequate knowledge was perceived as a barrier to effective nutrition care to the patient. Many beliefs and attitudes do not always translate into practice. Although the M-KAP of physicians and nurses is lower than in some other countries/studies, it highlights a strong need for more nutrition professionals in the hospital and increasing nutrition education to improve nutrition care in hospitals in Palestine. Furthermore, establishing a nutrition task force in hospitals elaborated by dietitians as the unique nutrition care provider will assure to implementation of a standardized nutrition care process.

**Supplementary Information:**

The online version contains supplementary material available at 10.1186/s41043-023-00355-9.

## Background

Nutritional care is a multidisciplinary responsibility of hospital staff, including managerial level, and its integration within healthcare workforce activities is essential [1]. Therefore, the nutrition care process (NCP) is a significant issue to dietetics professionals, and there are rising needs for implementation across the globe [2]. NCP refers to any interactive step-by-step pathway undertaken by a health professional and documented in the medical record to promote a patient's food-related behavior and subsequent health outcomes. NCP can be considered a problem-solving method and a systematic approach to the foundation of medical nutrition therapy, which can screen, assess, diagnose, treat, and evaluate nutrition-related problems and malnutrition-related processes [3]. As a result, poor nutrition care can cause harm or has the potential to cause harm to patients including malnutrition.

Malnutrition is prevalent globally, considered a burden on patients, families, hospitals, and the healthcare system, including economic burden [4]. European Society Of Parenteral and Enteral Nutrition (ESPEN) defines malnutrition seen in hospitalized patients as a combination of cachexia (disease-related) and malnutrition (inadequate consumption of nutrients) as opposed to malnutrition alone [5]. Thus, the diagnosis of malnutrition is based on a combination of at least one phenotype criterion (i.e., unintentional weight loss, low BMI, or reduced muscle mass) and one etiology criterion (i.e., reduced food intake, malabsorption, or severe disease with inflammation) [6, 7].

To avoid malnutrition, all healthcare providers, including hospital management, must work as one team [8]. For example, physicians are responsible for writing admission orders based on the present patient's condition, including food. Furthermore, nurses are the direct care staff in hospital wards who have the most day-to-day contacts with patients, and they frequently perform initial nutrition screening [9]. As such, they play critical roles in the continuing identification of patients at risk of malnutrition due to inadequate food consumption and in the administration of nutrition-supportive therapies to patients on their wards [10].

Quality of nutrition care is lacking in Palestine and considered a widespread challenge as many hospitalized patients are treated for many medical problems while having their nutritional needs ignored. To the best of our knowledge, no data were found on the prevalence of malnutrition in hospitalized patients and there is no previous research related to nutrition care in Palestine. Planning and formulating strategies and interventions necessitate a thorough understanding of what healthcare professionals know and practice in routine nutritional care and what personal factors and barriers affect nutrition practice and attitude. Although malnutrition can affect recovery and outcome in acute care patients [11], little is known about malnutrition in in Palestine and they are limited to non-hospitalized patients [12, 13] and even less is known about healthcare providers’ knowledge, attitudes, and practices (M-KAP) toward nutrition care in hospitalized patients.

This study was designed to assess the KAP of physicians and nurses toward quality measures of nutrition care in hospitalized patients as a developmental approach to improve the nutrition process and promote nutrition care plans in Palestine. Physicians and nurses were selected because they are considered as the vast majority of hospital staff, and the first healthcare provider that the patient comes in contact with despite that nutrition care is a multidisciplinary responsibility [14]. Additionally, the current study highlights the reasons for inadequate nutrition in hospitalized patients and share concerns about the importance of developing and directing change management strategies in hospital settings to complete the integrated cycle of quality of health care provided. The present research explores, for the first time in Palestine, the effect of measuring M-KAP of hospital staff in routine clinical care, as it is a useful method to provide valuable input to improve awareness of hospital staff, define staff responsibility, promote nutrition, identify focus areas, provide feedback and direction to optimize the nutrition care process and quality of care strategies [15].

## Methods

### Study design

This is a cross-sectional study. Data were collected between April 1, 2019, and June 31, 2019.

### Settings

This study collected data from physicians and nurses in two hospitals types: governmental (*n* = 5) and non-governmental (*n* = 4) hospitals in the North West Bank of Palestine.

### Sample size

Sample Size was calculated using the Raosoft sample size calculator. 5% margin of error with 95% confidence interval, 50% response distribution, and a population of 19,000 were used. The sample size calculated was 377. Eligible participants were nurses and physicians with clinical roles and direct patient contact in an inpatient department of the selected hospitals.

Participants were told about the study after satisfying sample selection criteria, and those who agreed to participate voluntarily were included in the sample.

### Population

**Fig. 1:**
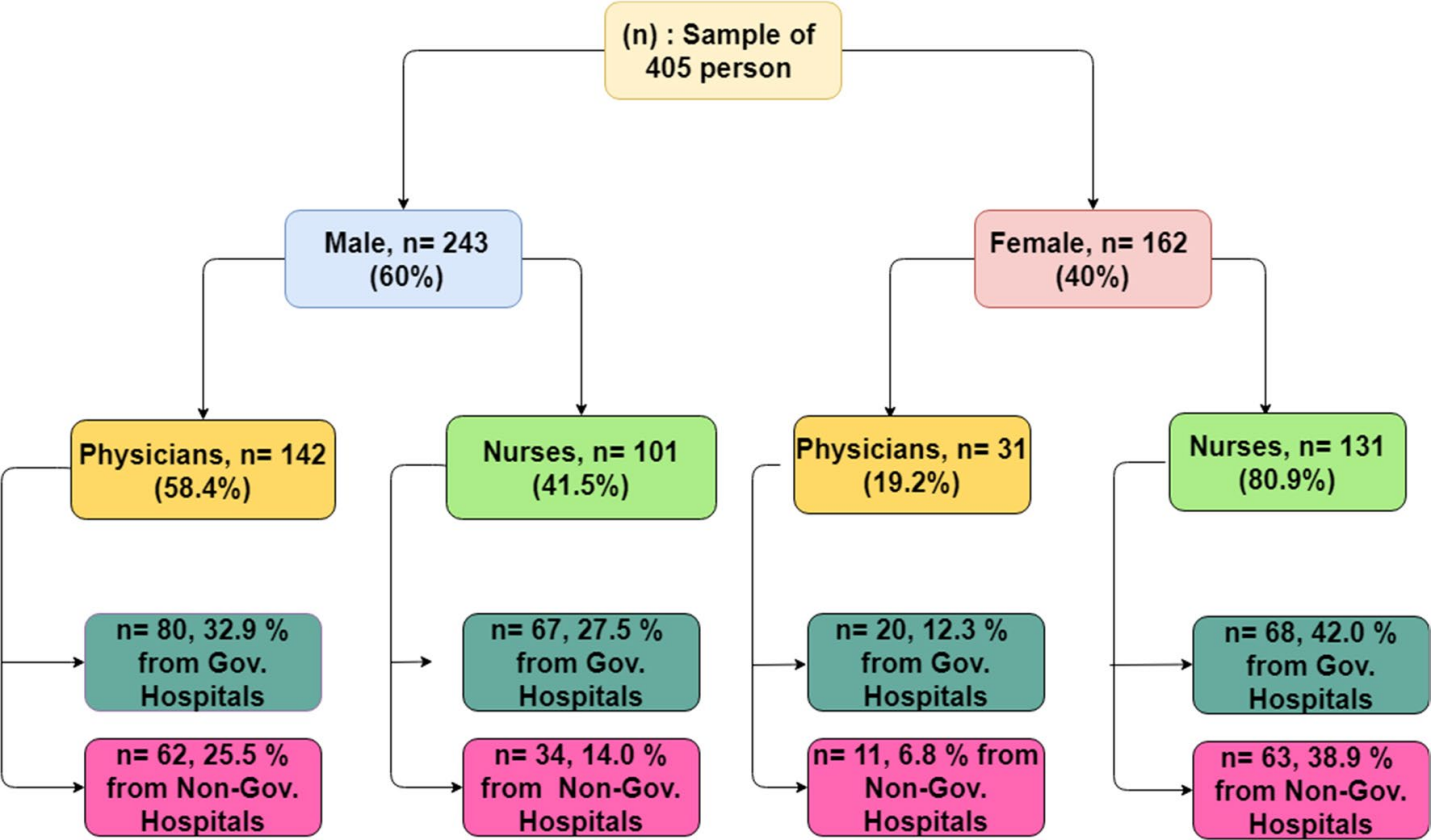
Selection and sample size

Four hundred and five nurses (practical nurses (who provide assistance to doctors or registered nurses), registered (who provide direct care to patients) and midwifery) and physicians (residents and specialists) from governmental and non-governmental hospitals in the North West- Bank-Palestine were selected by a convenience sample method. Subjects were recruited based on a nonrandom sample based on Fig. 1. Dietitians and ancillary services practitioners were excluded as too many questions were not relevant, and their results would not represent the general staff in the unit. In addition, dietitians would be aware and trained about nutrition care. Therefore, including their opinions may skew the data to have a more positive opinion about the importance of nutrition, compared to other staff.

The questionnaire was applicable for eligible participants from nurses and physicians with clinical roles and direct patient contact in any inpatient departments of the selected hospital in the North West Bank (Nablus, Tulkarem, Qalqelia, Jenin, and Tubas).

### Tool (data collection form)

A questionnaire, adapted from a previous study [15], was used after translating to the Arabic language and validated. The original tool was developed by the More-2-eat (M2E) project, which measured Malnutrition Knowledge, Attitude, and Practice (M-KAP). This tool was established in accordance with integrated nutrition pathway in a cute care (INPAC) to represent critical prevention, detection, and treatment efforts [15, 16]. In general, hospitals utilized this instrument as a baseline measure to highlight where they needed to enhance nutrition care and illustrate change over time when improvements were made [17–19].

In this study, no special permission was required from the developers to reuse any part of this questionnaire to measure nurses' and physicians' knowledge, attitude, and practice regarding malnutrition and nutrition care.

This questionnaire consisted of six parts:

- The first section included sociodemographic information such as the participant's age, gender, specialty, years of experience, type of hospital, and units they worked in.
- The second section consisted of 15 questions about the knowledge and perception of nurses and physicians concerning malnutrition and nutrition care. Scores were added from questions 1–15 to get the knowledge score (range 15–75).
- The third section consisted of 5 questions about attitude. Scores were added from questions 16–20 to get the attitude score (range 5–25). Scores were also added from questions 1–20 to get KA score (range 20–100).
- The fourth section consisted of 7 questions about nutrition care practice at the patient's bedside. In this section, scores were added from 21–27 to get the practice score (range 7–28).
- The fifth section investigated the perceptions regarding the most important reasons why patients may not eat in the hospital unit [10, 20]. Nine options were listed for the participants to choose from them.
- The sixth section investigated the perceptions regarding the most important reasons why patients may get insufficient nutrition support (tube feeding, artificial nutrition) [10, 20]. Again, nine options were listed for the participants to choose from them. The last sections (i.e., fifth and sixth sections) were shortened and slightly modified to make them relevant to Palestinian hospitals and staff. Concerning questions relating to the most important reasons why patients may not eat in the hospital unit and reasons why patients may get insufficient nutrition support, responses included “yes”, or “no”.

The questionnaire included five options for the knowledge and attitude part, ranging from "strongly disagree" to "strongly agree," as follows: strongly disagree = 1, somewhat disagree = 2, sometimes = 3, somewhat agree = 4, strongly agree = 5. The first, eighth, thirteenth, and fifteenth questions were reverse coded. Respondents had four alternatives in the practice section: "never," "sometimes," "often," and "always," with the practice score being "never = 1", "sometime = 2", "often = 3", and "always = 4". The overall KAP score was computed using questions 1–27 and the subscale total, with higher scores indicating more positive K, A, and P.

For the knowledge and attitude section, the questionnaire provided five choices ranging from 'strongly disagree' to 'strongly agree' as follows: strongly disagree = 1, somewhat disagree = 2, sometimes = 3, somewhat agree = 4, strongly agree = 5. Questions (1, 8, 13, and 15) were reverse coded. In the practice section, the respondents had 4 options: 'never'; 'sometimes'; 'often' and 'always', for the practice score: never = 1, sometimes = 2, often = 3, always = 4. While the total KAP score was obtained from questions 1–27, and the subscale total was calculated so that higher scores indicated more positive K, A, and P.

### Validity and reliability of the tool

- The original form of the questionnaire was translated and back-translated following World Health Organization guidelines [21]. The Arabic version can be found at the end of this manuscript (Additional file 1).
- A focus group panel involved ten qualified nurses and physicians who were meeting the inclusion criteria, reviewed and evaluated the final questions' face and content validity, assessed the definition of wards, medical terminology, clear sequences of statements where the aim, objectives, and tool discussed.
- Before conducting the study, a modified questionnaire was tested with a pilot sample of five physicians and five nurses; data from the pilot sample were not included in the analysis. The questionnaire was revised for clarity and ease of use, and no changes were recommended.
- Cronbach alpha was used to check consistency between questions and was found to be accepted as follows: knowledge (68%), attitude (67%), practice (80.5%), and KAP (77%).

### Data collection procedure

The questionnaire was self-administered. Each survey took ten minutes to complete. The researchers gave the participants some background information about the research project, and where necessary, they explained some of the questions in the questionnaire. Participants were given a consent form that explained the objective of the study and guaranteed confidentiality. Participants have the option of participating or not.

### Ethical consideration and human subjects’ protection

Permission for the study was obtained from the *Institutional Review Board (IRB) of An-Najah National University*, Ministry of Health, and hospitals included in the study, and any other authorities concerned. All procedures were carried out in accordance with the Helsinki Declaration's ethical standards. Participants were informed about the purpose and benefits of the research. All data have been collected with confidentiality. The IRB authorized the research protocol (including the verbal consent process) and did not need written consent because the research has no harm or physical risk to participants. Everyone who took part was notified that their data will be coded and anonymized.

### Statistical analysis

The IBM SPSS software version 23 was used to enter, clean, manage, and analyze data. Frequencies and means were computed for categorical and continuous data. Descriptive statistics were employed to determine the response frequency and describe the sample. According to data normality, KAP was shown as the entire mean, whereas median was shown as an individual for K, A, and P. For numerical variables, data were given as mean and standard deviation (SD) or median and interquartile range (IQR), and for nominal variables, the frequency with percentages. The Kolmogorov–Smirnov test was used to ensure that all results were normal. The independent sample *t*-test and ANOVA were used for data with a normal distribution. In contrast, for data with a non-normal distribution, the Mann–Whitney *U* test and the Kruskal–Wallis H test were used. The Pearson correlation coefficient was used to look into the possibility of a relationship between two continuous variables (malnutrition knowledge, attitude, and practice scores). Staff roles, specialization, type of hospital, units, and years of experience were all expected to impact K, A, and P and therefore the KAP scores; thus, samples were spread among units to see whether there were any connections. As needed, statistical tests to assess relationships and significance were employed. Significant was defined as a *p* value of less than 0.05.

## Results

### Sociodemographic data

Demographic information for the sample is presented in Table 1. A total of four hundred and thirteen questionnaires were collected from the governmental 235 (58.02%) and non-governmental hospitals (41.98%) in seven of the hospital units: surgical, internal, pediatric, gynecology and obstetrics, intensive care unit (ICU), emergency and other departments as follows (23.95%, 20.49%, 14.81%, 13.58%, 12.58%, 9.38%, 5.19%, respectively). Eight of the respondents were excluded from the results as they did not directly contact patients inwards. Respondents were mostly male (60.00%). The age of respondents was equally distributed between 21 and 69 years old, the mean age of the respondents was 32.77 ± 9.09 years, and the median was 30 years with an interquartile range of 27.0–36.0.

**Table 1:** Demographics data of the sample (*n* = 405)

Variable	Number (%)
*Gender*	
Male	243 (60.00)
Female	162 (40.00)
*Age categories (year)*	
< 30	194 (47.90)
30–39	135 (33.33)
40–49	45 (11.11)
50–59	24 (5.93)
≥ 60	6 (1.48)
*Type of hospital*	
Non-governmental	170 (41.98)
Governmental	235(58.02)
*Units*	
ICU	51(12.58)
Surgical	97(23.95)
Internal	83(20.49)
Gynecology and obstetric	55(13.58)
Pediatric	60(14.81)
Emergency	38(9.38)
Others	21 (5.19)
*Job title*	
Resident physician	109 (26.91)
Specialist physician	64 (15.80)
Practical nurse	49 (12.10)
Staff nurse/registered nurse	150 (37.04)
Nurse-midwife	33 (8.15)
*Contract type*	
Full time	375 (92.59)
Part-time	30 (7.41)
*Years of experience*	
< 3	89 (21.98)
03-Oct	202 (49.88)
> 10	114 (28.15)

Physicians (42.71%) and nurses (57. 29%) were asked to complete the survey. Two groups of physicians and three groups of nurses participated in the study: specialist physician (15.80%), practical nurse (12.10%), and nurse-midwife (8.15%), where most respondents were from registered nurses (37.04%) and resident physicians (26.91%). The majority (92.59%) was full-time contract type. Around half (49.88%) of the respondents had job experience between three to ten years.

### Knowledge of nurses and physicians for malnutrition and nutrition care

The median knowledge score of nurses and physicians for malnutrition and nutrition care is 53.00 with an interquartile range of 49.00–57.00. Both age and hospital's units showed a significant association with knowledge (*p* < 0.05) (Table 2). On the other hand, there was no significant association between gender, type of hospital, job title, and years of experience. Respondents in surgical, internal, pediatric, and ICU reported better knowledge, in the previous order, more so than other hospital units. Respondents in young and middle adulthood showed good knowledge than older adulthood. Knowledge increased in critical care units (*p* < 0.05).

**Table 2:** Association between sociodemographic factors and all domains of malnutrition knowledge, attitudes, and practices (M-KAP)

Variables	Number (%)	Knowledge score median (*Q*1–*Q*3)	Attitude score median (*Q*1–*Q*3)	Knowledge and attitude score	Practice score median (*Q*1–*Q*3)	KAP score
	*N* = 405			Median (*Q*1–*Q*3)		Mean (SD)
*Gender*						
Male	243 (60%)	54 (49.00–57.00)^$,a^	18 (16.00–19.00)^$,a^	71 (66.00–75.00)^$,a^	15 (13.00–17.00)^$,a^	85.59 (9.72)^$,b^
Female	162 (40%)	53 (49.00–56.00)	18 (15.00–20.00)	70 (65.00–75.00)	15 (13.00–18.00)	85.67 (9.18)
*Age categories*						
< 30	194 (47.90%)	54 (49.00–57.00)*^,c^	18 (15.00–20.00) *^,c^	71.00 (65.00–76.00) ^$,c^	15 (13.00–18.00) ^$,c^	86.10 (9.41) ^#,d^
30–39	135 (33.33%)	53 (49.00–57.00)	18 (15.00–19.00)	70.00 (66.00–75.00)	15 (13.00–18.00)	85.85 (9. 23)
40–49	45 (11.11%)	54 (50.00–56.00)	18 (17.00–20.00)	72.00 (68.00–75.00)	15 (13.50–16.50)	86. 22 (8.56)
50–59	24 (5.93%)	49 (45.5–54.75)	18 (16.00–19.00)	67.50 (64.00–73.75)	16 (13.00–17.00)	82.16 (9.01)
> 60	6 (1.48%)	43.5 (41.00–53.50)	19 (14.25–19.25)	62.50 (56.00–72.50)	11 (5.25–16.75)	73.83 (18. 23)
*Type of hospital*						
Non-governmental	170 (41.98%)	54 (49.00–57.00) ^$,a^	18.50 (16.00–20.00) ^#,a^	71.50 (67.00–76. 25) *^,a^	16 (13.00–19.00) *^,a^	86.95 (9.84) *^,b^
Governmental	235(58.02%)	53 (49.00–56.00)	17.00 (15.00–19.00)	70.00 (65.00–75.00)	15 (13.00–17.00)	84.66 (9.15)
*Units*						
ICU	51(12.58%)	55 (50.00–59.00) ^#,c^	17 (15.00–19.00) ^$,c^	71 (68.00–77.00) ^#,c^	18 (15.00–20.00) ^#,c^	89.07 (9.45) ^#,d^
Surgical	97(23.95%)	54 (48.50–57.00)	18 (16.00–20.00)	71 (65.00–76.00)	15 (13.00–17.00)	85. 23 (8.74)
Internal	83(20.49%)	54 (50.00–58.00)	18 (16.00–20.00)	73 (67.00–76.00)	15 (13.00–17.00)	87.06 (10.93)
Gynecology & obstetric	55(13.58%)	51 (48.00–54.00)	17 (14.00–19.00)	68 (63.00–73.00)	14 (10.00–16.00)	81.30 (9.75)
Pediatric	60(14.81%)	54 (50.00–58.00)	18 (17.00–20.00)	72 (67.00–76.00)	15 (12.00–17.00)	86.55 (8. 26)
Emergency	38(9.38%)	51 (49.00–54.25)	17 (16.00–19.00)	68 (65.00–72. 25)	15 (14.00–17.00)	83.94 (6.61)
Others	21 (5.19%)	52 (50.00–55.50)	17 (15.50–19.00)	70 (66.50–73.00)	16 (10.50–18.50)	85.09 (10.30)
*Job title*						
Resident physician	109 (26.91%)	54 (49.00–57.00) ^$,c^	18 (16.00–19.00) ^$,c^	72 (67.00–75.00) ^$,c^	14 (12.00–16.00) ^#,c^	84.77 (8. 22) ^#,d^
Specialist physician	64 (15.80%)	52 (49.00–54.00)	18 (16.00–19.00)	70 (67.00–73.00)	13 (11.00–15.00)	82.84 (8.34)
Practical nurse	49 (12.10%)	53 (49.50–55.00)	18 (15.00–20.00)	70 (64.00–75.50)	17 (15.00–20.00)	87.08 (8.84)
Staff nurse	150 (37.04%)	54 (49.00–58.00)	18 (15.00–20.00)	71 (65.00–76.50)	17 (14.00–19.00)	87.62 (10.80)
Nurse-midwife	33 (8.15%)	52 (48.50–54.00)	17 (13.50–19.00)	69 (63.00–72.50)	14 (12.00–17.00)	82.57 (8.03)
*Contract type*						
Full time	375 (92.59%)	53 (49.00–57.00) ^$,a^	18 (16.00–19.00) ^$,a^	71 (65.00–75.00) ^$,a^	15 (13.00–18.00) ^$,a^	85.50 (9.57) ^$,b^
Part time	30 (7.41%)	54 (49.75–57. 25)	18.50 (15.75–20.00)	70 (66.75–76.00)	16 (14.00–19.00)	87. 20 (8.61)
*Years of experience*						
< 3	89 (21.98%)	53 (49.00- 57.00) ^$,c^	18 (16.00–20.00) ^$,c^	70 (65.50–75.00) ^$,c^	14 (12.50–17.50) ^$,c^	85.43 (9.03) ^$,d^
03-Oct	202 (49.88%)	54 (49.00–57.00)	18 (15.00–19.00)	71 (66.00–76.00)	15 (13.00–18.00)	85.85 (9.68)
> 10	114 (28.15%)	52 (49.00–56.00)	18 (16.00–20.00)	71(65.00–75.00)	16 (13.50–17.50)	85.37 (9.60)

^$^, not Significant (*p* value ≥ 0.05); *, *p* value < 0.05; #, *p* value ≤ 0.01

^a^statistical significance of differences calculated using the Mann–Whiney U test

^b^statistical significance of differences calculated using the independent sample t-test

^c^statistical significance of differences calculated using the Kruskal–Wallis test

^d^statistical significance of differences calculated using the one-way ANOVA

In response to knowledge about malnutrition, almost half of those surveyed (56%) strongly agreed that nutrition is important. The patient's weight should be taken on admission (50.6%). In comparison, only a quarter of respondents (26.9%) believed that patients should be screened for malnutrition on admission, and only 19.8% believed that patient's weight should be taken on discharge. On the other hand, only 9.6% strongly agreed, and 39.3% somewhat agreed that malnutrition is a high priority in their hospitals; a quarter of respondents believed that malnutrition patients needed to follow up in the community after discharge (23.2%), and they are highly needed to be given an adequate amount of food in the hospital to enhance recovery (25.7%); Additional file 2: Table S1 summarizes participant’s knowledge responses in detail (Additional file 2: Table S1).

### Attitudes regarding malnutrition and nutrition care

The median attitudes score regarding malnutrition and nutrition care is 18.00 with an interquartile range of 16.00–20.00. A quarter of respondents perceived how to refer to a dietitian (23.2%), but a minority of respondents knew when to refer (13.1%) and when the patient was at risk or malnourished (11.9%). 9.6% of participants strongly indicated that they knew some strategies to support patients' food intake at mealtime, while 51.1% agreed that they need more training to better support the patients' nutrition needs. Table S2 summarizes participant’s attitude responses in detail (Additional file 2: Table S2).

Table 2 shows that the only significant association was between attitude and type of hospital (Mann–Whitney *U* test, *p*-value < 0.05). Respondents who worked in non-governmental hospitals reported a better attitude (median = 18.50) than those in governmental hospitals (median = 17.00). Gender, age, specialty, units, years of experience, and contract type did not significantly affect attitude.

Taken together, the results on knowledge and attitude showed that the median KA score is 71 with an interquartile range of 65.00–75.00. Table 2 illustrates the significant association between knowledge/attitude and two of the demographics: Types of hospitals (Mann–Whitney, *p* < 0.05) and units (Kruskal–Wallis test, *p* < 0.05). Respondents who worked in non-governmental hospitals reported better knowledge/attitude (median = 71.50) than those in a governmental hospital (median = 70.00). Respondents who worked in internal (median = 73), pediatric (median = 72), ICU (median = 71), surgical (median = 71), reported higher knowledge/attitude level than those who worked in other departments, gynecology and emergency (median = 70, 68, and 68, respectively).

### Practices regarding malnutrition and nutrition care

The median practices score regarding malnutrition and nutrition care is 15.00 with an interquartile range of 13.00–18.00. Surprisingly, a minority of respondents always provide adequate nutrition care to the patient at the bedside during mealtime; the most striking observation to emerge is that only 14.6% of responders have been realigned their tasks, so they do not give interruption the patient at meal time. On the other hand, 16.8% of respondents check whether the patient has all that he needs to eat, only 8.4% of respondents help a patient with opening food packages, and 9.9% assist the patient in eating if he needs help, while almost 5% visits and check patients during their mealtime to see how well they are eating and give encouragement to a patient's family to bring food from home for the patient is permitted. Only 7.7% of the respondents provided malnourished patients nutrition education material on discharge. Additional file 2: Table S3 summarizes participant’s practice responses in detail (Additional file 2: Table S3).

The results, as shown in Table 2, indicate that the type of hospital was significantly associated with practice toward nutrition care at the bedside with *p* < 0.05 (Mann–Whitney *U* test) in addition to specialty and hospital's units were significantly associated with it (Kruskal–Wallis test, *p* < 0.05). Other demographics did not significantly associate with practices like gender, age, and years of experience. Higher practice on nutrition care was detected in non-governmental hospitals (median = 16) than governmental hospitals (median = 15). Staff and practical nurse participants reported higher practice than resident doctors, nurse midwives, and specialist doctors (median = 14, 14, and 13, respectively). ICU participants reported higher practice (median = 18) than other hospital units with significant differences.

### Knowledge, attitude, and practice (KAP) regarding malnutrition and nutrition care

Overall, these results indicate that the mean KAP score was 85.62 ± 9.50, with a minimum of 45 and a maximum of 113. Table 2 presents an overview of the statistically significant association between sociodemographic data and total KAP. No statistical difference has been shown between sexes, years of experience, and respondents' contract type. Table 2 illustrates that age, specialty, and units were significantly associated with knowledge, attitude, and practice toward nutrition care (one-way ANOVA, *p* < 0.05) in addition to the type of hospital was significantly associated with it (independent sample t-test, *p* < 0.05). Respondents from non-governmental hospitals reported higher scores (mean = 86.95) more than governmental hospital participants (mean = 84.66). Respondents in adulthood groups (< 30, 30–39, and 40–49 years old) reported higher KAP score (mean = 86.10, 85.85, and 86.22, respectively) more than older adulthood groups (50–59 and above 60 years old) (mean = 82.16, 73.83, respectively). Respondents in the ICU units reported higher KAP score (mean = 89.07) followed by internal unit (mean = 87.06), pediatric unit (mean = 86.55), and surgical unit (mean = 85.23), other departments (mean = 85.09), emergency (mean = 83.94), and gynecology and obstetrics unit (mean = 81.30). Staff and practical nurse participants reported higher KAP scores (mean = 87.62, 87.08) followed by resident doctors (mean = 84.77), specialist doctors (mean = 82.84), and nurse-midwife (mean = 82.57).

### The correlations between knowledge, attitude, and practice scores regarding the quality of nutrition care

Between respondents' knowledge and attitude scores, there was a significant moderate positive correlation (*r* = 0.134, *p* < 0.001). According to the findings, individuals with greater knowledge were more likely to affect nutrition care positively. There was a significant moderate positive correlation between respondents' knowledge and practice scores (*r* = 0.273, *p* < 0.001). These findings suggest that individuals with high understanding were more likely to conduct good nutrition care. Knowledge/attitude and practice had a modest but significant positive association (*r* = 0.348, *p* < 0.001). According to the findings, respondents with excellent knowledge/attitude were more likely to have good nutrition care practices. There was a modest but significant positive connection between respondents' attitudes and practice ratings for nutrition care (*r* = 0.266, *p* < 0.001), indicating that those with a positive attitude are more likely to have more practice (Table 3).

**Table 3:** Correlations between knowledge, attitude, and practice

Correlations	Pearson correlation	*p*-value
Knowledge/attitude	0.134	0.007
Knowledge/practice	0. 273	0.001
Knowledge, attitude/practice	0.348	0.001
Attitude/practice	0. 266	0.001

### Barriers to adequate in-hospital nutrition and nutrition support

**Fig. 2:**
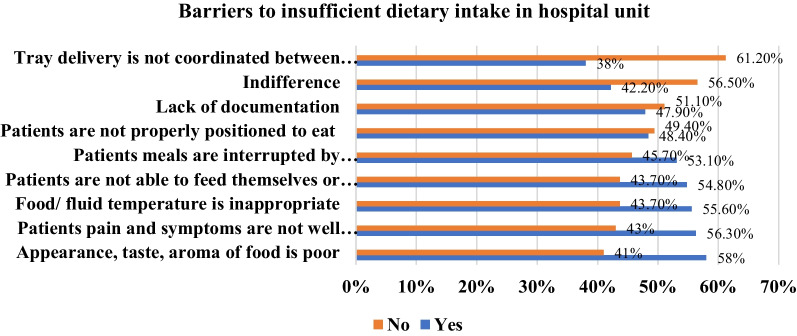
The most important barriers why patients may not eat in hospital unit

The results also indicated that almost half of the respondents believe that the most important barriers to inadequate intake of food are related to food appearance, taste and aroma of meals served (58.0%), patient medical condition (56.3%), the temperature of meals (55.6%), patients need an assistant at mealtime (54.8%), interruption during the mealtime (53.1%), patients are not well-positioned to eat (48.4%), lack of documentation (47.9%), and 38.0% of respondents referred the reason to miscoordination of tray delivery between foodservice and nursing. However, the most surprising barrier was the indifference to having patients’ adequate food intake (42.2%); (Fig. 2).

**Fig. 3:**
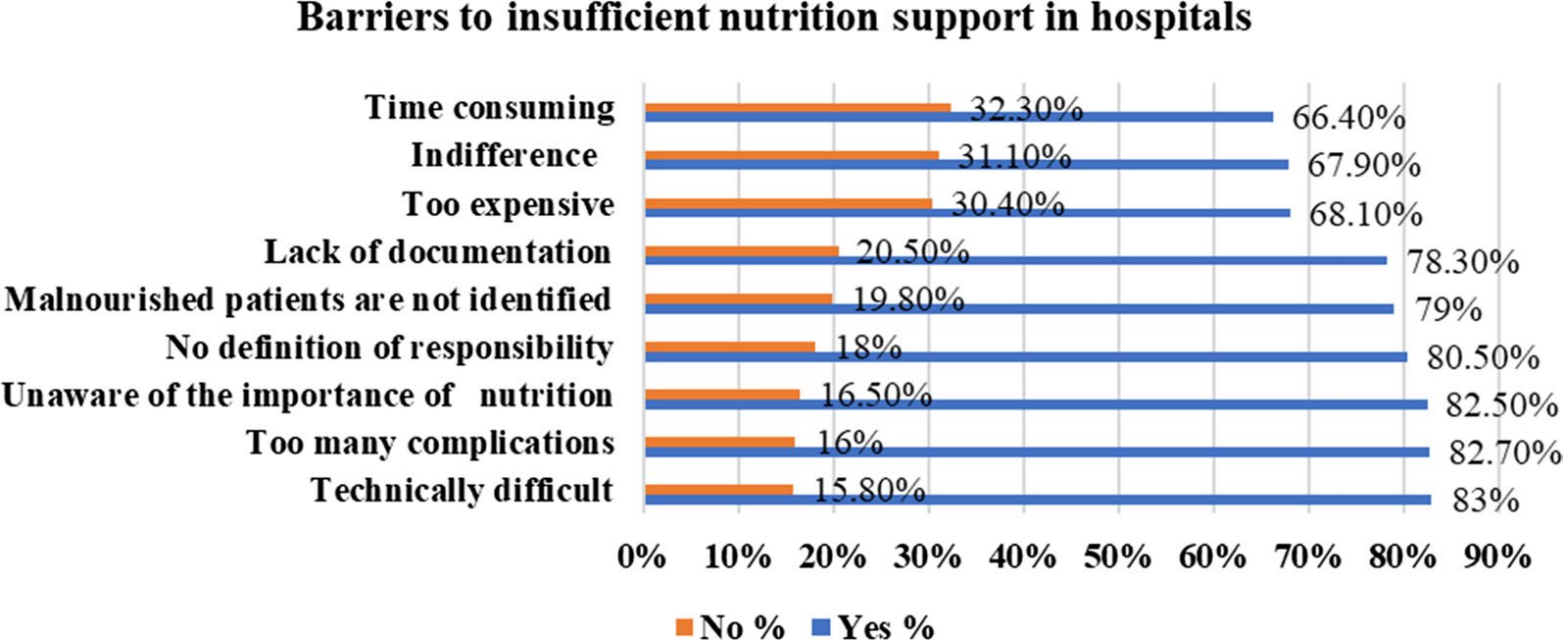
The most important barriers why patients may get insufficient nutrition support (tube feeding, artificial nutrition)

On the other hand, the research has touched on the reasons for insufficient nutrition support in hospitalized patients. The results indicated that most of the respondents believed that the most important reasons related to the technically difficult issues; for example, proximal GI obstruction, multiple upper abdominal operations and gastrectomy that may affect insertion (83.0%), complications like catheter removal and hyperglycemia (82.7%), unaware of the importance of nutrition (82.5%), no clear definition of the job description (80.5%), malnourished patients are not identified (79.0%), lack of documentation (78.3%), too expensive (68.1%), indifference (67.9%) and time-consuming (66.4%); (Fig. 3).

## Discussion

This is the first study to offer some important information on malnutrition and the quality of nutrition care services for patients in hospital settings in Palestine. The patient's nutritional status is still not considered a medical priority despite numerous advances in clinical care. The importance and originality of this study, which evaluates the level of knowledge, attitude, and practices related to malnutrition and nutrition treatment among Palestinian nurses and physicians working in hospitals and see whether they were at acceptable levels. The findings of this work may contribute to the field of nutrition management systems in clinical care practice.

Unfortunately, as indicated in the literature review, no studies have been conducted to evaluate physicians' and nurses' knowledge, attitudes, and behaviors on nutrition treatment and malnutrition among hospitalized patients in Palestine. Moreover, nutrition care in hospitals has received little attention in Palestine due to the gradual effects of nutrition. Common barriers include lack of nutrition knowledge among healthcare providers, lack of clearly defined nutrition responsibilities in planning and managing nutrition care, and lack of nutrition specialists in hospitals. To date, four of the nine hospitals in this study do not include nutrition specialists among their staff. Furthermore, only one hospital from the ones mentioned above screened patients for any possible risk indicator of malnutrition.

Malnutrition is associated with negative outcomes for patients, including increased risk of immune suppression [22], higher infection and complicated rate, increased muscle loss [23], increased risk of pressure ulcer, and impaired wound healing [22], longer hospital stay, higher treatment costs and increased morbidity and mortality [22, 24–29]. To address hospital malnutrition, the More-2-Eat implementation project (M2E) has developed from the year 2015 to 2017 an evidence-based integrated nutrition pathway in a cute care (INPAC); to guide all healthcare staff in the prevention, detection, and treatment of malnutrition in medical and surgical patients and to support practice improvement from direct care staff to policy management level [16, 30]. Furthermore, meal service to the patient is an integral part of nutrition care. Improving meal intake and minimizing barriers to inadequate food intake are essential and relevant to the patient and hospital outcomes [10, 11, 20]. Therefore, poor nutrition care pathways can cause decreased patient satisfaction, which may, in turn, lead to decreased food consumption, unintended weight loss, and other complications [31, 32].

### Knowledge, attitude and practice of nurses and physicians concerning malnutrition and nutrition care

This study showed that the quality of nutrition care at hospitals is in the early stage; the result has shown that approximately half of the respondents (56.0%) strongly agreed that nutrition is important for the patient's recovery and management of the disease. The result is lower than a similar study that reported that most respondents (88%) strongly agreed that nutrition is important [15]. Practical nurses and other health professionals, including general practitioners, have shown similar views in other studies where they perceived that nutrition is important for chronic disease management and supported best practice guidelines (Australian Governmental Department of Health and Aging 2003) to improve nutrition care for the management of patients with chronic diseases [33–35].

Malnutrition is a common and highly prevalent condition among patients in acute hospital settings [36]. However, it continues to be an underdiagnosed and largely under-recognized health problem in many hospital settings [37–40]. This study confirms the previous findings since only 9.6% of respondents strongly believed malnutrition is a high priority. Most respondents (79.0%) said that a barrier to nutrition care was that malnourished patients were not identified. Screening all patients for malnutrition is essential to identify patients at risk of malnutrition and develop a care plan [41]. However, in this study, only 26.9% strongly agreed that all patients should be screened; furthermore, only one of nine hospitals has a nutrition policy and screening tool for malnutrition. In comparison, half of the respondents (50.6%) strongly agreed that patient's weight should be taken on admission, and only 19.8% strongly agreed that patient's weight is necessary at discharge. Results are less than similar research that has been conducted in Canada, which reported the results with the above-mentioned dependent variables as follows (20%, 49%, 69%, and 28%), respectively [15]. This might be due to lack of hospital nutrition policy, lack of nutrition knowledge, difficulty identifying patients at nutritional risk as supported by previous research [42], and the absence of dietitians in addition to evidence-based screening and assessment tools as a key first step for best practice in Palestine.

It is worth mentioning that respondents showed lower mean scores toward questions related to nutrition care responsibility than other related questions in the questionnaire. This finding contradicts the previous study, which has suggested that nutrition care is multidisciplinary responsibility [14]. Our study reported that 30.6% of respondents believed that nutrition care is the only role of a dietitian, and most of them (78%) believed that malnutrition patients should have an individualized treatment by a dietitian but only 23% knew how and only 13% knew when. Only 38.7% of the respondents agreed that all staff involved in patient care could help set up the tray, and 45.75% of the respondents agreed that they could provide hands-on assistance to eat when necessary. Some of the hospitals have a dietitian. This finding demonstrates the need for more dietitians/nutrition professionals in hospitals along with the need for education about how and when to refer to a dietitian.

In our study, the mean KAP score was 85.62 ± 9.50, with a minimum of 45 and a maximum of 113, which seems to be less than similar research, which found that the score was (93.6/128) (range: 51–124). This finding may be translated to a lower perceived and actual quality of nutrition care [15].

This research revealed a significantly meaningful positive correlation between nutrition knowledge, attitude, and practice regarding nutrition care in hospitals. The result is consistent with a previous Croatian study published in 2018 that showed a statistically significant difference in the median number of positive attitudes of general practitioners based on additional education in nutrition and the implementation of nutrition care practice in everyday work with patients [43]. The KAP described here are essential for providing successful nutritional care in malnourished patients, and improving these factors may result in improved patient outcomes. These results are in line with previous research which found that the KAP questionnaire significantly enhanced after the implementation of the malnutrition screening tool [19, 44, 45].

Even though the correlations between knowledge, attitude, and practice were all positive and statistically significant in this study, unfortunately, many beliefs and attitudes did not always translate into practice. For example, several studies in a systematic review study published in 2013 reported a conflict between nurses' theoretical recognition and actual implementation of nutrition guidelines [46, 47]. This study is consistent with previous studies [46, 47] that found most respondents (76.1%) agreed that giving malnourished patients adequate food will enhance their recovery. However, only 4.9% visit and check a patient during their mealtime to see how well they are eating. In addition, 60.5% agreed that interruption during mealtime could negatively affect food intake, and only 14.6% realign their task, so they do not interrupt a patient during mealtime.

Considering the nutrition field is an interesting and appreciated field in the hospital, the results confirmed that lack of nutrition knowledge is a barrier to insufficient and inappropriate nutritional practice. It was observed from several lines of evidence that increased knowledge level will lead to an increase in examined patients and detection of malnutrition [42, 48]. As a result, there is a high need for training courses to improve knowledge, attitudes, and practice regarding nutrition care in hospitals. Nurses were more likely to feel positive about nutrition care as a part of their responsibilities after receiving recent professional training in the field [49].

### Factors affecting knowledge, attitude, and practice

All nurse respondents were ward nurses rather than from the other nursing positions, and more than half of the 232 nurses were female (56.4%). Thus, the results seem close to other research with a similar representation from academic and community hospitals [10].

A study has shown a significant relationship between age categories and knowledge and total KAP score, similar to other studies that found a significant relationship between nurses' age and level of nutrition knowledge. Those older nurses show higher average knowledge scores [50, 51]. In contrast, younger participants showed higher median and mean scores than the other older ones in this study. This could be due to the emerging higher education support system both at school and universities that shed light on the importance of nutrition care.

Types of hospitals in which respondents worked were not significantly associated with nutrition knowledge. This might be because all staff came almost from the same educational level. On the contrary, there was a significant association between types of hospital and attitude, knowledge/attitude, practice, and total KAP score. Non-governmental hospitals show better knowledge/attitude, practice, and total KAP score than governmental hospitals. This might be due to continuous training, dietitians being involved in nutrition care, and the presence of nutrition policy and available screening tools [19, 44, 45, 52].

In this study, there was a significant correlation between the respondents' units and the level of nutrition knowledge, knowledge/attitude, practice score, and total KAP scores. In addition, the ICU unit was obtained the highest mean and median score, similar to a study conducted in the Middle East, which revealed that ICU nurses scored higher than internal medicine nurses toward knowledge and perceived quality of nutrition care [53]. This might be due to nutrition self-course or awareness due to a sense of responsibility toward high-risk patients in the ICU. Therefore, their nutrition status is heavily dependent on what the healthcare providers know and behave to achieve a higher level of nutrition care [54, 55].

It is worth mentioning that a significant relationship was found between the specialty of the respondents and practice in addition to the total KAP score. Practical and staff nurses showed a higher score than the physician did. This result verifies previous findings that ensuring optimal nutrition care depends heavily on nurses who play a pivotal role in ensuring adequate nutritional care is delivered to the patient at the bedside [53].

On the other hand, there was no significant difference in total KAP score for years of practice, similar to previous findings [15, 48, 56]. Furthermore, it was reported that no significant difference between years of nursing experience and clinical nutrition knowledge (*p* = 0.827). This may confirm that education is better than clinical experience in the case of nutrition care.

### Barriers to adequate in-hospital nutrition and nutrition support

Hospitalized patients, regardless of their BMI, may suffer from malnutrition because of reduced dietary intake due to illness-induced poor appetite, gastrointestinal symptoms, reduced ability to chew or swallow, or patients have missed meals due to interruptions or investigation, and nothing by mouth (NPO) status for diagnostic and therapeutic procedures [37]. On the other hand, adequate food and energy intake was an important factor determining LOS and patient clinical status [57, 58]. However, this was not always done in practice, and energy goals were frequently not met due to many barriers related to insufficient nutritional intake at the patient bedside. In this study, similar results have been found. For example, the lowest score was obtained for nutrition practice at the bedside (55.32%) compared to knowledge and attitude scores (71.8%, 68.2%), respectively. In contrast, many barriers affect sufficient dietary intake and nutrition support at the bedside. For example, the research revealed that the most important barriers to inadequate intake were related to food quality at the bedside, i.e., food appearance (58.0%). In comparison, illness effects on food intake (56.3%), patients were unable to open packages/unwrapping (54.8%) and meals interrupted by staff (53.1%). These results were equal to the most common barriers to insufficient food intake in the surgical and medical units of 18 Canadian hospitals of acute care but from patient’s point of view [11]. On the contrary, lack of awareness, lack of experience in critical care (technically difficult with too many complications), resource constraints such as time and money were the most common barriers for insufficient nutrition support, similar to a Canadian study in the ICU [59]. In addition, inadequate knowledge and confidence were seen as barriers to providing patients with appropriate nutrition treatment [60].

Results confirmed a high need for training courses to improve the knowledge and practice of nutrition care in hospitals as many beliefs and attitudes did not always translate into practice. In addition, low staff priority to nutrition care due to lack of time, many jobs to do, and the task are not relevant have been reported in much previous research and is highly needed for further study.

The absence of nutrition care has an impact on both patients and staff. Patients' nutritional requirements were ignored while being treated for medical problems. Furthermore, most patients are unaware of the critical role that good nutrition plays in their treatment and recovery from illness. Patients in need of nutrition therapy were unaware of the appropriate diet and texture of the provided food corresponding to their medical condition and the potential food–drug interactions that could jeopardize their medical status. As a result, dietary education and patient information should be given top importance in educational efforts at all levels. Unfortunately, only 7.7% of responders in this research give nutrition instruction materials to malnourished patients. Even though a Cochrane review of 36 studies published in 2008 examined the evidence surrounding dietary advice and nutritional intake of adults with illness-related malnutrition, the findings compared a combination of dietary advice, dietary supplements, or no advice with outcome measures. They concluded that dietary advice with nutritional supplements might be more effective than advice alone or no advice [61].

### Strengths and limitations

This study is the first in Palestine to evaluate knowledge, attitudes, and practice levels regarding nutrition care for healthcare providers in hospital settings. It shed light on the importance of a standardized nutrition care process to manage malnutrition and increase the quality of nutrition care. In addition, the diversity of respondents included different healthcare sectors.

The most important limitation lies in the fact that the data were obtained through a self-administered questionnaire. The respondents may react to being well educated, and the work environment is well suited to nutrition care. Therefore, results could overestimate the attitude and practice score due to recall bias. It is worth mentioning that the questionnaire asked questions related to perceptions of nurses and physicians and self-perceived attitudes and practices and may not be representative of what occurs in real life, the actual barriers, or their significance. In addition, the analyzed results from the snapshot timing may not be representative. To investigate the effect of healthcare provider training and education on nutritional status, attitudes and behaviors needed to be analyzed over time [62, 63]. The convenience of sample methods may have limited the generalizability of the current study.

## Conclusions

The main goal of the current study was to evaluate knowledge, attitude, and practices regarding malnutrition and quality of care in addition to the most important staff perceptions of patient barriers to food intake and/or insufficient nutrition support in hospital settings, North Palestine. This study showed that the respondents generally had low nutritional KAP scores. Inadequate knowledge was perceived to be a barrier to effective nutrition care to the patient. In addition, many beliefs and attitudes do not always translate into practice. Therefore, barriers to effective nutrition care must be followed by the administration managers. It is recommended that hospitals establish a nutrition task force based on NCP or INPAC pathway that can engage and improve the nutrition care process for patients during their stay from admission to discharge [10, 20, 64–66]. Availability of high-quality documentation of the nutrition care process is essential from the moment of the patient's admission to the ward to the time of discharge, especially since recognizing malnutrition in hospitalized patients is not often a priority in clinical practice in Palestine. Additionally, nutrition knowledge is necessary to improve nutrition practice, but nutrition knowledge seems insufficient to change practice in routine clinical care. Furthermore, encouraging changes in the system to increase nutrition education among hospital staff, encourage implementation of nutrition screening tools, and increase the presence of dietitians in the hospitals.

## Supplementary Information

**Additional file 1**. The Arabic version of Knowledge, Attitude and Practice scale for measuring Quality of Nutrition Care in Hospitals.

**Additional file 2**. **Table S1**: Distribution of responses to each knowledge question with a five-point Likert scale ranked from 1 to 5 (Strongly disagree, disagree, not sure, agree, and strongly agree). **Table S2**: Distribution of responses to each attitude question with a five-point Likert scale ranked from 1 to 5 (Strongly disagree, disagree, not sure, agree, and strongly agree). **Table S3**: Distribution of responses to each practice question with a five-point Likert scale ranked from 1 to 4 (Never, sometimes, often, and always).

## Data Availability

The data from our surveillance are not available on the public domain due to privacy and ethical restrictions, but anyone interested in using the data for scientific purposes is free to request permission from the corresponding author. This manuscript forms part of a Master graduation project submitted to An-Najah National University and the full version of thesis was published as part of self-archiving in institutional repositories (that is, university repository: https://repository.najah.edu/handle/20.500.11888/16714?show=full).

## Abbreviations

ESPEN: European Society for Parenteral and Enteral Nutrition
ANOVA: Analysis of variance
BMI: Body mass index
IRB: Institutional review board
ICU: Intensive care unit
INPAC: Integrated Nutrition Pathway in Acute Care
M-KAP: Malnutrition knowledge, attitude, practice
LOS: Length of stay
M2E: More-to-eat
NCP: Nutrition care process
NOURISH: Nutrition effect on unplanned readmission and survival in hospitalized patients
NPO: Nothing by mouth
NNUH: An-Najah National University Hospital
SPSS: Statistical Package of Social Sciences
SD: Standard deviation
