# Supplementary material for: Healthcare providers’ knowledge, attitude, and practice on quality of nutrition care in hospitals from a developing country: a multicenter experience
Source: J Health Popul Nutr. 2023 Mar 7;42:15. doi: 10.1186/s41043-023-00355-9 (PMC9990276; doi:10.1186/s41043-023-00355-9)
Supplement: Supplementary file 1 — Additional file 1. The Arabic version of Knowledge, Attitude and Practice scale for measuring Quality of Nutrition Care in Hospitals. [file 41043_2023_355_MOESM1_ESM.docx]

**Additional file 1** The Arabic version of Knowledge, Attitude and Practice scale for measuring Quality of Nutrition Care in Hospitals

| **يرجى تقييم معرفتك لكل عبارة من العبارات التالية** | | **موافق بشدة** | **موافق الى حد ما** | **أحيانا** | **لا أوافق** | **لا اوافق نهائيا** |
| --- | --- | --- | --- | --- | --- | --- |
| 1 | التغذية ليست مهمة لتعافي المرضى في المستشفى |  |  |  |  |  |
| 2 | يجب عمل مسح للكشف عن سوء التغذية للمرضى عند الدخول |  |  |  |  |  |
| 3 | يجب أخذ وزن المريض عند الدخول |  |  |  |  |  |
| 4 | يمكن لجميع الموظفين المشاركين في رعاية المرضى المساعدة في إعداد صينية الوجبة الوجبة وفتحها وإحضار اللازم ليتمكن المريض من تناول طعامه |  |  |  |  |  |
| 5 | يمكن لجميع الموظفين المشاركين في رعاية المرضى تقديم المساعدة في عملية تناول الطعام للمرضى عند الضرورة |  |  |  |  |  |
| 6 | يعطي سوء التغذية أولوية عالية في المستشفى |  |  |  |  |  |
| 7 | إن إعطاء المرضى الذين يعانون من سوء التغذية كمية كافية من الطعام سيعزز من شفائهم |  |  |  |  |  |
| 8 | يحتاج جميع المرضى الذين يعانون من سوء التغذية إلى علاج فردي من قبل اختصاصي تغذية |  |  |  |  |  |
| 9 | لدي دور مهم في نعزيز مدخول المريض الغذائي |  |  |  |  |  |
| 10 | يعتبر رصد كمية الطعام وسيلة جيدة لتحديد الحالة التغذوية للمريض |  |  |  |  |  |
| 11 | يمكن أن تؤثر المقاطعات أثناء الوجبة تأثيراً سلبياً على المدخول الغذائي للمريض |  |  |  |  |  |
| 12 | إن تشجيع تناول الطعام للمريض هو وظيفة كل موظف |  |  |  |  |  |
| 13 | الرعاية الغذائية للمريض مقتصره على أخصائي التغذية * |  |  |  |  |  |
| 14 | يحتاج المرضى الذين يعانون من سوء التغذية الى متابعة في المجتمع بعد الخروج |  |  |  |  |  |
| 15 | وزن المريض غير ضروري عند الخروج |  |  |  |  |  |

| **يرجى تقييم مواقفك مع كل عبارة من العبارات التالية** | | **موافق بشدة** | **موافق الى حد ما** | **أحيانا** | **لا أوافق** | **لا اوافق نهائيا** |
| --- | --- | --- | --- | --- | --- | --- |
| 16 | انا أعرف متى بالإمكان الرجوع الى أخصائي تغذية |  |  |  |  |  |
| 17 | اعرف كيف الوصول الى اخصائي التغذية |  |  |  |  |  |
| 18 | أعرف متى يكون المريض عرضة لسوء التغذية أو يعاني من سوء التغذية |  |  |  |  |  |
| 19 | أنا أعرف بعض الاستراتيجيات لدعم تناول الطعام في وجبات الطعام |  |  |  |  |  |
| 20 | أحتاج إلى مزيد من التدريب لدعم احتياجات التغذية لمرضاي بشكل أفضل |  |  |  |  |  |

| **يرجى تقييم معدل القيام بما يلي** | | **ابدا** | **احيانا** | **غالبا** | **دائما** |
| --- | --- | --- | --- | --- | --- |
| 1 | تتحقق من أن المريض لديه كل ما يحتاجه لتناول الطعام  (مثل أطقم الأسنان والنظارات) |  |  |  |  |
| 2 | تقوم بمساعدة المريض في قتح وجبته |  |  |  |  |
| 3 | تقوم بمساعدة المريض في تناول طعامه عند الحاجه |  |  |  |  |
| 4 | عند سماح إحضار الوجبة، تشجه المريض على إحضار الوجبة للمريض من المنزل |  |  |  |  |
| 5 | تقوم بزيارة المريض خلال وقت الطعام لمعرفه مدى |  |  |  |  |
| 6 | أقوم بإعادة تنظيم مهماتي حتى لا أقوم بمقاطعة المريض أثناء وقت الوجبة |  |  |  |  |
| 7 | يتم تزويد المريض بنشره غذائية عند الخروج |  |  |  |  |
