# Supplementary material for: Healthcare providers’ knowledge, attitude, and practice on quality of nutrition care in hospitals from a developing country: a multicenter experience
Source: J Health Popul Nutr. 2023 Mar 7;42:15. doi: 10.1186/s41043-023-00355-9 (PMC9990276; doi:10.1186/s41043-023-00355-9)
Supplement: Supplementary file 2 — Additional file 2. Table S1: Distribution of responses to each knowledge question with a five-point Likert scale ranked from 1 to 5 (Strongly disagree, disagree, not sure, agree, and strongly agree). Table S2: Distribution of responses to each attitude question with a five-point Likert scale ranked from 1 to 5 (Strongly disagree, disagree, not sure, agree, and strongly agree). Table S3: Distribution of responses to each practice question with a five-point Likert scale ranked from 1 to 4 (Never, sometimes, often, and always). [file 41043_2023_355_MOESM2_ESM.docx]

**Additional file 2:** **Table S1.** Distribution of responses to each knowledge question with a five-point Likert scale ranked from 1 to 5 (Strongly disagree, disagree, not sure, agree, and strongly agree). **Table S2.** Distribution of responses to each attitude question with a five-point Likert scale ranked from 1 to 5 (Strongly disagree, disagree, not sure, agree, and strongly agree). **Table S3.** Distribution of responses to each practice question with a five-point Likert scale ranked from 1 to 4 (Never, sometimes, often, and always).

**Table S1** Proportion of responses for knowledge questions (N=405)

| **Question no. #*** | **Question statement** | **Strongly agree (%)** | **Somewhat agree (%)** | **Sometimes**  **(%)** | **Somewhat disagree (%)** | **Strongly disagree**  **(%)** |
| --- | --- | --- | --- | --- | --- | --- |
|  | Please your rate agreement with the following statements | | | | | |
| 1 | Nutrition is not important to a patient’s recovery in hospital* | 7(1.7) | 11(2.7) | 27(6.7) | 133(32.8) | **227(56.0)** |
| 2 | All patients should be screened for malnutrition at admission to hospital | **109(26.9)** | 185(45.7) | 87(21.5) | 17(4.2) | 7(1.7) |
| 3 | A patient’s weight should be taken at admission | **205(50.6)** | 148(36.5) | 42(10.4) | 3(.7) | 3(.7) |
| 4 | All staff involved in patient care can help set up the meal tray, open packages etc. | **35(8.6)** | 122(30.1) | 113(27.9) | 104(25.7) | 29(7.2) |
| 5 | All staff involved in patient care can provide hands-on assistance to eat when necessary | **31(7.7)** | 154(38) | 126(31.1) | 78(19.3) | 15(3.7) |
| 6 | Malnutrition is a high priority at this hospital | **39(9.6)** | 159(39.3) | 129(31.9) | 60(14.8) | 13(3.2) |
| 7 | Giving malnourished patients an adequate amount of food will enhance their recovery | **104(25.7)** | 204(50.4) | 74(18.3) | 17(4. 2) | 1(.2) |
| 8 | All malnourished patients require individualized treatment by a dietitian * | 116(28.6) | 200(49.4) | 57(14.1) | **21(5. 2)** | **3(.7)** |
| 9 | I have an important role in promoting a patient’s food intake | **34(8.4)** | 195(48.1) | 140(34.6) | 23(5.7) | 9(2. 2) |
| 10 | Monitoring food intake is a good way to determine a patient’s nutritional status | **47(11.6)** | 222 (54.8) | 106(26. 2) | 25(6. 2) | 2 (.5) |
| 11 | Interruptions during the meal can negatively affect patient food intake | **38(9.4)** | 207(51.1) | 120(29.6) | 31(7.7) | 4(1.0) |
| 12 | Promoting food intake to a patient is every staff member’s job | **49(12.1)** | 157(38.8) | 91(22.5) | 84(20.7) | 19(4.7) |
| 13 | Nutritional care of a patient is only the role of the dietitian* | 34(8.4) | 90(22. 2) | 94(23.2) | **166(41.0)** | **19(4.7)** |
| 14 | Malnourished patients who are discharged need follow up in the community | **94(23.2)** | 227(56.0) | 66(16.3) | 13(3.2) | 3(.7) |
| 15 | A patient’s weight is not necessary at discharge* | 14(3.5) | 49(12.1) | 84(20.7) | 177(43.7) | **80(19.8)** |
|  | Total knowledge score (out of 75) |  |  |  |  |  |

**Table S2** Proportion of responses for attitude questions (N=405)

| **Question no. #*** | **Question statement** | **Strongly agree (%)** | **Somewhat agree (%)** | **Sometimes**  **(%)** | **Somewhat disagree (%)** | **Strongly disagree**  **(%)** |
| --- | --- | --- | --- | --- | --- | --- |
| 16 | I always know when to refer to a dietitian | **53(13.1)** | 183(45.2) | 113(27.9) | 39(9.6) | 16(4.0) |
| 17 | I know how to refer to a dietitian | **94(23.2)** | 150(37.0) | 84(20.7) | 50(12.3) | 26(6.4) |
| 18 | I know when a patient is at risk of malnutrition or is malnourished | **48(11.9)** | 201(49.6) | 120(29.6) | 22(5.4) | 13(3.2) |
| 19 | I know some strategies to support food intake at meals | **39(9.6)** | 151(37.3) | 141(34.8) | 57(14.1) | 14(3.5) |
| 20 | I need more training to better support the nutrition needs of my patients | **40(9.9)** | 167 (41.2) | 141(34.8) | 37(9.1) | 20(4.9) |
|  | Total attitude score (out of 25) |  |  |  |  |  |
|  | Total KA score (out of 100) |  |  |  |  |  |

*: These are negative questions and the scoring was reversed: Strongly Disagree (5); Somewhat Disagree (4); sometimes (3); Somewhat Agree (2); Strongly Agree (1); Blank (0). A higher score indicates more knowledge/ attitude. For example, in the first question 1, 4.38/5 means that more people think that nutrition is important. For question 8, 2/5 means that more people believe that all malnourished patients require individualized treatment by a dietitian.

**Table S3** Proportion of responses for practice questions from questions (N=405)

| **Question no #*** | **Question statement** | **Never** | **Sometimes** | **Often** | **Always** |
| --- | --- | --- | --- | --- | --- |
|  |  |  |  |  |  |
| 21 | Check the patient has all that they need to eat (e.g. dentures, glasses) | 47(11.6) | 183(45.2) | 103(25.4) | **68(16.8)** |
| 22 | Help a patient with opening food packages | 80(19.8) | 216(53.3) | 66(16.3) | **34(8.4)** |
| 23 | Assist a patient to eat if they need help | 69(17.0) | 221(54.6) | 70(17.3) | **40(9.9)** |
| 24 | If permitted, encourage a patient’s family to bring food from home for the patient | 90(22.2) | 209(51.6) | 75(18.5) | **23(5.7)** |
| 25 | Visit and check a patient during their meal time to see how well they are eating | 82(20.2) | 237(58.5) | 59(14.6) | **20(4.9)** |
| 26 | Realign my tasks so I do not interrupt a patient during their meal time | 33(8.1) | 169(41.7) | 137(33.8) | **59(14.6)** |
| 27 | At discharge of a malnourished patient, provide the patient or family with nutrition education material | 117(28.9) | 188(46.4) | 55(13.6) | **31(7.7)** |
|  | Total practice score (out of 28) |  |  |  |  |
|  | Total KAP score (out of 128) |  |  |  |  |

# These questions were adapted from Laur C, Marcus H, Ray S, Keller H: **Quality Nutrition Care: Measuring Hospital Staff's Knowledge, Attitudes, and Practices**. *Healthcare (Basel, Switzerland)* 2016, **4**(4):79.

* Permission to use this instrument to measure Malnutrition Knowledge, Attitude, and Practice (M-KAP) in this study was obtained from the developers of the questionnaire Dr. Heather Keller
